# Miniaturised dual-modality all-optical ultrasound probe for laser interstitial thermal therapy (LITT) monitoring

**DOI:** 10.1364/BOE.494892

**Published:** 2023-06-20

**Authors:** Shaoyan Zhang, Semyon Bodian, Edward Z. Zhang, Paul C. Beard, Sacha Noimark, Adrien E. Desjardins, Richard J. Colchester

**Affiliations:** 1 Department of Medical Physics and Biomedical Engineering, University College London, Malet Place Engineering Building, London WC1E 6BT, UK; 2 Wellcome/EPSRC Centre for Interventional and Surgical Sciences, Charles Bell House, University College London, 43-45 Foley Street, London W1W 7TY, UK; 3 Materials Chemistry Centre, Department of Chemistry, University College London, 20 Gordon Street, London WC1H 0AJ, UK

## Abstract

All-optical ultrasound (OpUS) has emerged as an imaging paradigm well-suited to minimally invasive imaging due to its ability to provide high resolution imaging from miniaturised fibre optic devices. Here, we report a fibre optic device capable of concurrent laser interstitial thermal therapy (LITT) and real-time *in situ* all-optical ultrasound imaging for lesion monitoring. The device comprised three optical fibres: one each for ultrasound transmission, reception and thermal therapy light delivery. This device had a total lateral dimension of <1 mm and was integrated into a medical needle. Simultaneous LITT and monitoring were performed on *ex vivo* lamb kidney with lesion depth tracked using M-mode OpUS imaging. Using one set of laser energy parameters for LITT (5 W, 60 s), the lesion depth varied from 3.3 mm to 8.3 mm. In all cases, the full lesion depth could be visualised and measured with the OpUS images and there was a good statistical agreement with stereomicroscope images acquired after ablation (t=1.36, p=0.18). This work demonstrates the feasibility and potential of OpUS to guide LITT in tumour resection.

## 1. Introduction

Recent years have seen an increase in minimally invasive procedures to treat diseases, such as cancer. One such case is renal cell carcinoma (RCC), which represents about 5% of newly diagnosed cancer cases in the western world and the detection rate has been increasing over the last decade [1,2]. The most commonly diagnosed RCCs were classified in T1a stage with small dimensions, typically causing symptoms including local pain and metastatic spread [3]. Where feasible, partial nephrectomy is largely preferable to radical nephrectomy in order to preserve kidney function [2]. For this therapeutic strategy, image-guided ablation therapy in a minimally invasive manner has been increasingly used for small RCCs and is proving to be a safe and effective approach. The ablation can be carried out in a variety of ways, including radiofrequency ablation (RFA), cryoablation (CRA), microwave ablation (MWA), and laser interstitial thermal therapy (LITT). Currently, RFA and CRA are the most commonly used modalities for treating small tumours ( <5 cm) [2]. However, LITT, which delivers light via optical fibres with small lateral dimensions and high mechanical flexibility, is preferred for treating deep targets in the body [4]. In addition, the minimal invasiveness of LITT makes it more suitable for patients at high risk of complications due to pre-existing comorbidities [5].

The LITT procedure requires image guidance to avoid under- and over-treatment, which can result in the need for follow-up procedures and damage to surrounding healthy tissue, respectively. However, image guidance for LITT is challenging and current technologies present several limitations. Magnetic resonance (MR) thermometry can be used to provide temperature mapping during the LITT procedure, but, this technique is constrained by machine availability, procedural cost and low frame rate for real-time monitoring [6]. As an alternative, ultrasound thermometry and imaging have been utilised for ablation monitoring due to their low cost and simplicity. However, the low imaging resolution of conventional ultrasound probes limits the accuracy of ablation guidance [7].

All-optical ultrasound (OpUS) has emerged as a new imaging paradigm for minimally invasive surgery. OpUS involves the generation and reception of ultrasound waves using light, instead of the piezoelectric effect used in conventional ultrasound transducers [8–10]. OpUS can provide broad transmission bandwidth and high reception sensitivity for promising imaging resolution and tissue penetration depth during interventions [11,12]. In addition, the use of optical fibres enables highly miniaturised devices that can be incorporated into medical catheters and needles [11]. Further, the OpUS devices use all-optical components which are immune to electromagnetic interference and thereby compatible with MRI scanners [13]. Moreover, due to the use of low-cost materials and facile fabrication processes, it is well-suited for single-use devices.

With OpUS, ultrasound waves are generated optically via the photoacoustic effect. When pulsed or modulated light is absorbed within a photoacoustic material, the subsequent temperature rise in the material leads to a corresponding pressure rise which propagates as ultrasound waves [14]. For photoacoustic materials, polydimethylsiloxane (PDMS) composites are broadly used due to their high thermal expansion coefficient which is associated with high-pressure generation [8]. Since the PDMS composites are optically transparent for commonly used laser wavelengths, optical absorbing materials have to be incorporated. Several materials have been proposed in previous works, including carbon nanotubes [15,16], carbon black [17], organic dyes [18–20], metallic nanoparticles [21,22], quantum dots [23], graphene [24] and candle soot [25,26]. For ultrasound reception, resonance-based acoustic sensors on the optical fibre tips are typically used to detect ultrasound reflections [27]. Among these sensors, plano-concave resonators with high detection sensitivity and broad detection bandwidth, which can detect the acoustically induced deformations of its polymer cavity, are well-suited for minimally invasive procedures [28]. In previous works, the OpUS devices have been used successfully to achieve two- and three-dimensional imaging on *ex vivo* tissue [12,29,30], as well as *in vivo* real-time imaging [11,31]. Additionally, side-viewing devices have been developed for rotational imaging [32,33] and two-dimensional imaging in which the transmitter and receiver were combined onto a single optical fibre for further miniaturisation [14,34].

One study has demonstrated the use of OpUS for imaging during radiofrequency ablation [35]. However, this study used a benchtop imaging system which was too large for interventional applications. To overcome these constraints, we previously reported a fibre optic OpUS device for real-time M-mode imaging of surface tissue ablation [20]. This device allowed for tracking of lesions formed on the surface of the tissue. However, the lesions created were small ( <2 mm total depth) and the effect of an intermediate tissue layer was not explored. To monitor subsurface tissue ablation, as generated with LITT, further work was needed.

In this work, we developed a miniaturised all-optical device (lateral dimension <1 mm) integrated within a medical needle that can be inserted into tissue for simultaneous interstitial laser ablation and OpUS imaging. The all-optical device comprised three optical fibres; one for ultrasound transmission, one for ultrasound reception and one for delivering light for laser ablation. The device was tested on *ex vivo* lamb kidney tissue to demonstrate its capability for concurrently performing treatment and lesion monitoring. Additionally, the depth of the ablated lesion formed was tracked on M-mode OpUS images using an algorithm based on image segmentation. The lesion depth measured on OpUS was compared with visual inspections of microscope images obtained post-ablation.

## 2. Methods

### 2.1 Dual-modality fibre optic LITT and OpUS system

**Fig. 1.**
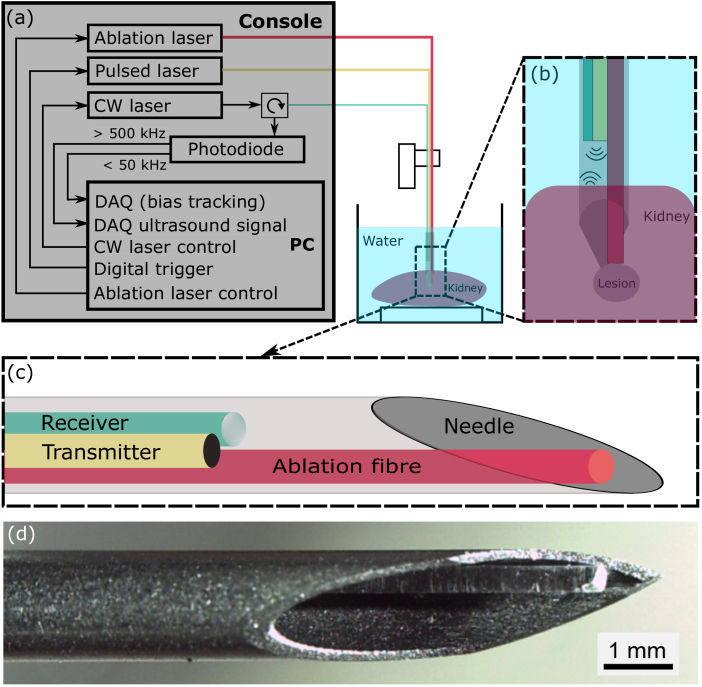
Schematic overview of the dual-modality OpUS imaging and LITT system including the console, fibre device and experiment setup. (a) Schematic of the console. (b) The set up of the fibre device for concurrent OpUS imaging and LITT. (c) Schematic of the fibre device; (d) the corresponding microscope image of the fibre optic device. The OpUS imaging device was recessed inside the needle lumen, out of view. CW laser: continuous wave laser; DAQ: Data acquisition card.

The dual-modality laser interstitial thermal therapy system comprised two parts; a fibre optic device for LITT delivery and ultrasound imaging, and a console to control and interrogate the fibre optic device (Fig. 1). The fibre optic device comprised three optical fibres; one to generate the outgoing ultrasound wave, one to receive the reflected ultrasound signals, and one to deliver light for LITT (Fig. 1 (c)).

The optical fibre for ultrasound transmission had a 400 µm core diameter and its distal end face was coated with a composite consisting of candle-soot nanoparticles (CSNPs) and PDMS. The detailed process for synthesising the composite and applying it to the fibre end face was described in previous work [26,36]. In brief, the PDMS (Sylgard 184 , Dow Corning, Europe) used was prepared by manually mixing the base and curing agent together in a 10 : 1 volumetric ratio, followed by degassing under a vacuum for 3 minutes. A step-index multi-mode fibre (WF 400 / 440 / 470 P, CeramOptec, Germany) with a 400 µm core diameter and a polyimide coating was placed 2.5 mm above the candlewick, with its cleaved distal tip facing the wick, and held there for 4 seconds. Subsequently, the fibre coated with CSNPs was manually dipped into the prepared PDMS solution, forming a bottom-up CSNP-PDMS bilayer composite coating on the fibre end face. The coatings were left for 24 hours in ambient conditions to cure. The fabricated transmitter generated ultrasound pressures up to 3 MPa at 1.5 mm from the coating, with corresponding pulse bandwidth of >30 MHz [26].

The optical ultrasound receiver comprised a plano-concave microresonator on the distal tip of a single mode optical fibre. The design and fabrication details were described in previous work [28,37]. In brief, a dielectric mirror with a reflectivity of 98 % was deposited onto the end face of a single mode optical fibre, the fibre was subsequently dip coated with an epoxy dome, followed by the deposition of another dielectric mirror ( 99 % reflectivity). Subsequently, a Parylene C overcoat was added to the fibre distal end for protection. The reflected ultrasound wave impinging on the plano-concave microresonator caused a change in the cavity thickness and therefore the reflectivity. These resonators provided a high detection sensitivity (noise-equivalent pressures of 2.1 mPa per $\sqrt{Hz}$ ), a broad detection bandwidth ( 40 MHz), a high level of miniaturisation ( 135 µm o.d at the distal end), and omnidirectional response [28].

The ablation fibre used a 400 µm core diameter optical fibre (WF 400 / 440 / 470 P, CeramOptec, Germany). The distal end was cleaved perpendicular to the optical fibre axis to form a uniformly flat end face for ablation light emission. The light was emitted ahead of the end face of ablation fibre with a beam divergence of 12.7 °, as defined by the numerical aperture of the fibre ( 0.22 NA).

The console used to control and interrogate the fibre device comprised three components (Fig. 1 (a)). For ultrasound excitation, a Q-swtiched Nd:YAG laser (wavelength: 1064 nm, pulse width: 2 ns, repetition rate: 100 Hz, pulse energy: 25 µJ, corresponding fluence: 20 ${\mathrm{mJ/cm}}^{2}$ , SPOT- 10 - 500 - 1064 , Elforlight, UK) was coupled directly into the ultrasound generation fibre. For the ultrasound reception, the plano-concave microresonator was interrogated using a system previously described [20]. In brief, a continuous wave (CW) tuneable laser (wavelength: 1500 - 1600 nm, power: 4.5 mW, Tunics T 100 S-HP CL, Yenista Optics, France) was coupled to the optical ultrasound receiver via a circulator. The reflected signal from the microresonator was detected with a custom photoreceiver, which split the signal into low frequency ( <50 kHz) and high frequency ( >500 kHz) components. The low frequency part was used to record the plano-concave microresonator transfer function and to estimate the optimum bias point. The high frequency part was used to record the modulation of reflected optical power induced by the cavity thickness change due to the incident acoustic wave, by which the reflected ultrasound signals were derived. For ablation, a diode laser (Axcel Photonics Inc, US) delivering 808 nm wavelength CW light was coupled into the LITT fibre and used to heat the tissue during the experiments. The output power and firing duration of laser light were controlled by a laser driver (LDP-CW 18 - 5 , PicoLAS, Germany) and a custom LabVIEW programme (National Instruments, USA).

With the all-optical fibre device for simultaneous LITT and ultrasound imaging, the ultrasound transmitter, ultrasound receiver and ablation fibre were held together using heat shrink tube ( 1.02 mm internal diameter prior to shrinking, polyethylene terephthalate, Nordson MEDICAL, USA). The distal end faces of the ultrasound transmitter and receiver were aligned at their distal tips, whilst the ablation fibre was advanced 10 mm to prevent heating of the receiver and allow the OpUS imaging probe to remain outside of the tissue for imaging. The OpUS device was integrated into a medical needle with a bevelled tip ( 16 G, length: 40 mm) where the end face of the ablation fibre was aligned with the bevel surface whilst the transmitter and receiver remained inside the needle lumen (Fig. 1 (c)). The device was mounted on a motorised stage (MTS 50 /M-Z 8 , Thorlabs, UK) and submerged in a water bath for experiments.

### 2.2 Concurrent LITT and OpUS imaging

The LITT and simultaneous OpUS lesion monitoring were performed on *ex vivo* lamb kidney. The lamb kidney was obtained from the animal slaughtered on the same day of the experiment and kept in the fridge ( 5 °C) prior to the experiment. During the experiment, the lamb kidney was affixed onto a plastic base using a cyanoacrylate adhesive and placed in the water bath. The medical needle carrying the OpUS device was inserted into the kidney tissue such that the distal needle tip was *ca.* 7 mm below the tissue surface. CW ablation laser light with a power of 5 W and duration of 60 s was used for LITT. At the same time as LITT, all-optical M-mode ultrasound imaging was performed to monitor lesion formation and progression in depth. The axial resolution of M-mode imaging can reach 40 µm [26] and lateral resolution is related to the aperture of the transmitter and divergence of the ultrasound wave [11].

For M-mode imaging, a custom LabVIEW programme was used for data acquisition, real-time image display and saving of the data for off-line processing. The acquired ultrasound A-lines were band-pass filtered ( 1.5−40 MHz) to suppress noise. Subsequently, the cross-talk signals due to the direct propagation between the surface of ultrasound transmitter and receiver were reduced by a general linear model [12]. In addition, the general linear model can reduce the signals from ultrasound reverberations within needle lumen and also non-ablated tissue, where the signals were unchanged over time, thus highlighting the lesion formation. Finally, the signals were Hilbert transformed to obtain the envelope, followed by a log transform to compress the dynamic range for display. To form the M-mode image, the individual A-lines were concatenated in time sequence to show temporal tissue changes. During the experiment, the M-mode ultrasound imaging was turned on 10 s prior to the start of LITT and continued for 10 s after the completion of LITT. This was to observe the tissue changes during the LITT procedure. The concurrent LITT and M-mode ultrasound imaging was repeated 19 times in different positions of the tissue. On the completion of each LITT procedure, the tissue was sliced through the centre of the lesion to enable lesion size measurement by the steremicroscopy (Leica, UK). It has been shown that during heating tissue echogenicity changes due to stiffness changes and bubble formation [38,39]. This is apparent in the ultrasound images as areas of increased brightness. Therefore, the lesion depth measured with OpUS was based on the segmentation of regions with increased brightness on grayscale ultrasound images, using the fuzzy-c means (FCM) algorithm [40,41] proposed in previous work [20]. In brief, the FCM algorithm assigned each pixel to different clusters by using a membership function that represented the probability of each pixel belonging to a specific cluster. The membership functions were updated iteratively to minimise the cost function. Following the convergence of the cost function, each pixel was assigned to the particular cluster with the greatest membership value. Subsequently, the morphological operators including image opening and closing were applied for extracting the ablation region. For the stereomicroscope lesion images, the depths were determined by averaging the visual inspection of three human observers, according to the colour change of the lesion caused by hyperthermia. After obtaining the OpUS and stereomicroscope measurements, the paired t-test (two-tailed, p <0.05 ) was performed to evaluate the accuracy of lesion depth measurement by OpUS.

### 2.3 *Ex vivo* kidney B-mode OpUS imaging pre- and post-ablation

To compare the efficacy of lesion visualisation between M-mode and B-mode OpUS imaging, a 2 D B-mode OpUS image of the *ex vivo* lamb kidney tissue was acquired pre- and post-LITT. The same OpUS device as used for concurrent LITT monitoring was used, however, the outer needle housing was removed for B-mode image acquisition, providing a large angular response. The OpUS device is capable of B-mode imaging with high resolution (axial: 40 µm, lateral: 200 µm) [26]. To acquire the B-mode images, the OpUS probe was translated laterally above the kidney surface in a stepwise fashion using a motorised translation stage. A step size of 50 µm and a total of 600 steps was used, to provide an image aperture of 30 mm. To form the OpUS images, the acquired pulse-echo signals were band-pass filtered (Butterworth, 1.5 - 40 MHz, $4^{\text{th}}$ order) for noise suppression, and then processed by time gain compensation (TGC) to reduce the effect of wave attenuation, followed by the cross-talk removal. After preprocessing, synthetic aperture reconstruction was carried out using a k-space method [42]. Finally, a Hilbert transform was applied to the image to extract the signal envelope, followed by a log transform for display.

## 3. Results

The device developed in this work comprised three optical fibres, one each for ultrasound transmission, ultrasound reception and delivery of light for laser therapy. The fibres were contained within a 16 G needle, yielding a total device outer diameter of 1.6 mm. The needle with this size has been used for clinical kidney LITT in previous work [43]. The use of optical fibres provided a high level of miniaturisation and mechanical flexibility, allowing the ease of medical needle or catheter integration, which is crucial for minimally invasive procedures. The ultrasound transmitter received excitation light from an optical fibre with an aperture of 400 µm; its end face was coated with a composite material that comprised candle-soot nanoparticles and PDMS. The aperture size offered low beam divergence [24] which has three advantages: good lateral resolution for M-mode imaging, increased penetration depth, and a reduction in ringing caused by generated ultrasound waves impinging on the inner wall of the needle. The CSNP-PDMS coating generated ultrasound waves with a peak-to-peak pressure of 3 MPa at 1.5 mm from the transmitter coating, which provided high tissue penetration and good signal-to-noise ratio.

### 3.1 Concurrent OpUS M-mode imaging and LITT

**Fig. 2.**
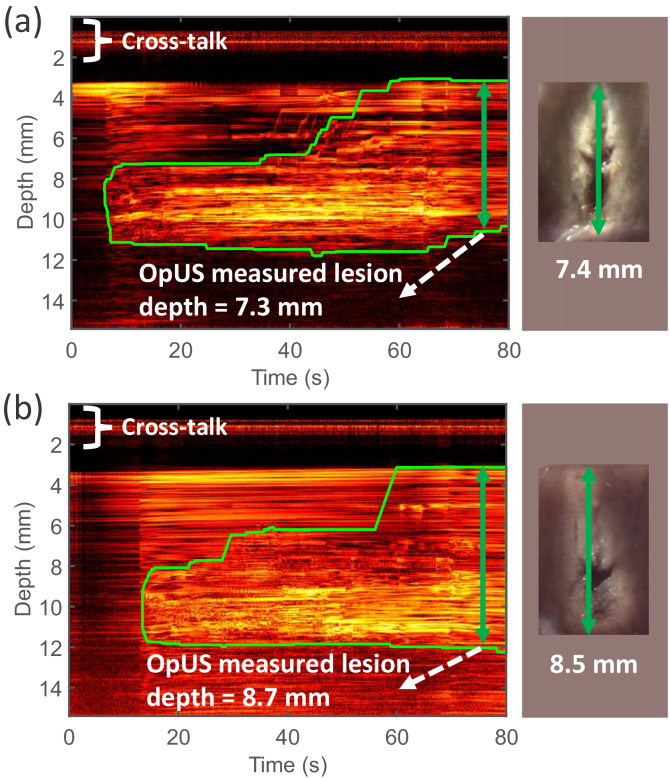
M-mode OpUS images of LITT procedures in which the lesion formed along the length of the needle. The depth axis of the M-mode image has been cropped for display purposes as ablation depths did not exceed those shown. The vertical length of the green boundaries indicates the ablation depth at different times. The cross-talk residual was shown at the top of the image. Inset: Corresponding microscope images of the cross-section of the resulting lesion.

**Fig. 3.**
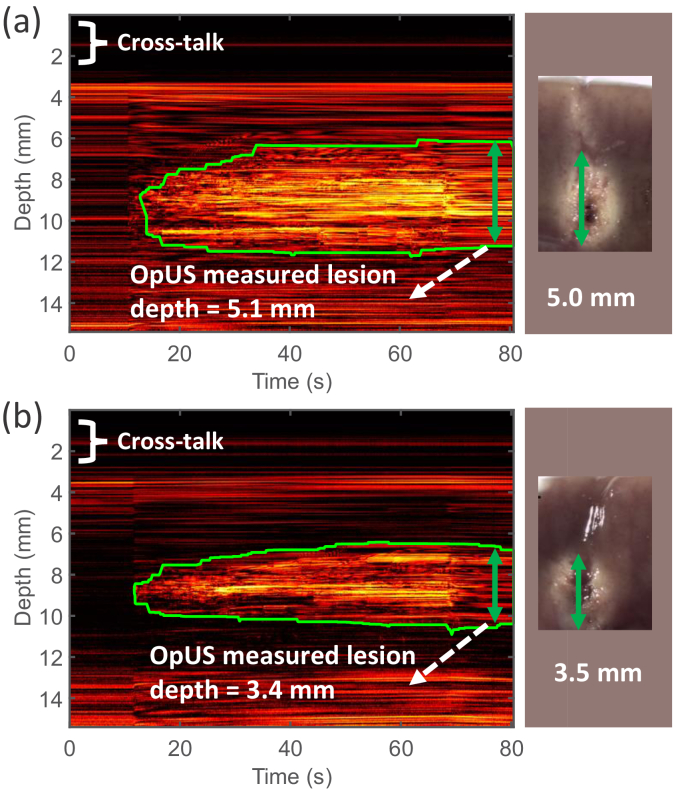
M-mode OpUS images of LITT procedures in which the lesion formed around the ablation fibre tip. The depth axis of the M-mode image has been cropped for display purposes as ablation depths did not exceed those shown. The vertical length of the green boundaries indicates the ablation depth at different times. The cross-talk residual was shown at the top of the image. Inset: Corresponding microscope images of the cross-section of the resulting lesion.

M-mode OpUS imaging allowed visualisation of the tissue throughout the procedure, where the tissue surface was delineated as a bright boundary (Fig. 2, 3). When the ablation laser was switched on (around 10 s on the M-mode image) the tissue contrast changed immediately, indicating the changes in tissue echogenicity induced by laser therapy. In all cases, throughout the ablation period, the area of contrast change grew bidirectionally from the optical fibre tip (positioned around 7 mm underneath the tissue surface) in the vertical dimension. This was apparent in M-mode OpUS images as an area of increasing brightness and changing contrast (Fig. 2, 3). During LITT experiments, two distinct types of lesions were observed; lesions which only formed around the ablation fibre tip (Fig. 3), and lesions which formed along the length of the needle used to house the probe (Fig. 2). This was confirmed with the microscope cross-sections taken after the experiments (Fig. 2, 3 inset).

**Fig. 4.**
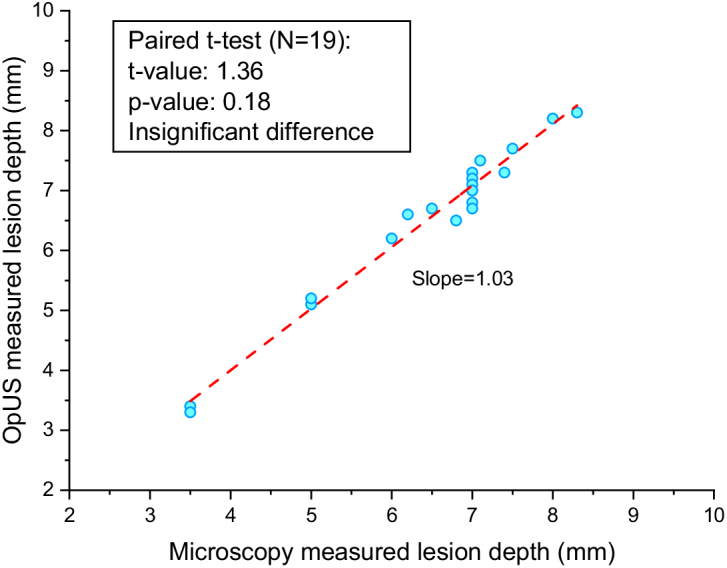
Correlation between lesion depths as measured with OpUS and stereomicroscopy.

The lesion area, corresponding to a region of increased brightness on the OpUS M-mode image, was tracked using the segmentation method described in the previous work [20], and was displayed as a green boundary. The resulting lesion depth was recorded after switching off the laser, where the contrast was stable. Lesion size as measured from the OpUS images varied across LITT procedures, even with fixed laser power and duration, ranging from 3.3 mm to 8.3 mm (Fig. 4). These OpUS measurements were confirmed with microscope cross-sections and demonstrated good agreement. The slope of the linear fit between the OpUS and microscopic measurements of lesion depth was found (Fig. 4) and a paired t-test was performed on the entire data set. The results indicated the difference between the lesion depth measurements by OpUS and microscope was statistically insignificant (slope= 1.03 , t= 1.36 , p= 0.18 ), showing the accuracy and efficacy of lesion monitoring by OpUS.

### 3.2 B-mode OpUS image of ex vivo lamb kidney

**Fig. 5.**
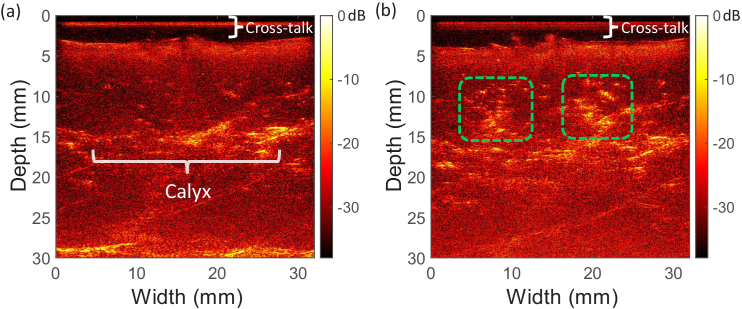
B-mode OpUS images of *ex vivo* kidney tissue, acquired (a) pre-ablation, (b) post-ablation. Dashed green boxes highlight the LITT lesions.

B-mode OpUS imaging provided an imaging depth >25 mm and high imaging resolution (axial: 40 µm, lateral: 200 µm [26]) on *ex vivo* lamb kidney (Fig. 5). The calyx was visualised around 10 mm below the kidney surface, with a distinct boundary (Fig. 5 (a)). The dynamic range of the pre- and post-ablation OpUS images was 35 dB, measured within the calyx at a depth of 15 mm (Fig. 5). Compared to the pre-ablation OpUS image, the region treated by LITT (green dashed boxes) exhibited increased brightness on the post-ablation image, indicating the formation of lesions (Fig. 5 (b)).

## 4. Discussion and conclusion

In this work, we present a miniaturised dual-modality fibre optic device that can be incorporated into a medical needle to perform LITT and ultrasound imaging simultaneously. Compared to the previous study [20], which demonstrated tracking of ablation lesions on the tissue surface, here we overcome significant challenges, demonstrating accurate tracking of subsurface lesions with depths up to 1 cm using a device built into a medical needle. To demonstrate the efficacy of this device for LITT monitoring during kidney tumour treatment, *ex vivo* lamb kidney tissue was used for experiments. During the study, one single laser output ( 5 W, 60 s) was chosen according to the clinical setup for laser tumour ablation used in previous work [44]. However, in this study, it was found that the lesion depths varied widely, from 3.3 mm to 8.3 mm despite the consistent laser input parameters. Additionally, there was variation in the lesion morphologies: in some cases, the lesion formed primarily around the distal tip of the ablation fibre with a spherical distribution (Fig. 3); in others, the lesion progressed along the needle, resulting in an elongated lesion (Fig. 2). This elongated distribution may have been caused by thermal conduction along the needle body. This uncertainty in lesion size is likely to be even greater *in vivo* due to variable conditions such as device positioning, presence of blood flow and varying tissue properties. These results motivate the need for real-time monitoring, to provide feedback to the operator and allow for LITT to be adjusted in real-time [45]. In the experiments carried out here, depth tracking via the OpUS image was successful in all cases, demonstrating its potential to provide this feedback. Further, the high correlation between the OpUS measured depths and those from stereomicroscopy supports this modality’s accuracy. The maximum lesion depth tracked by M-mode imaging was 8.3 mm, which was constrained by the size of the formed lesion during the LITT procedures. The presence of contrast at greater depths in both the M-mode and B-mode images obtained here suggests that larger lesions could be visualised. Follow-up studies could be used to confirm this, either using different LITT laser parameters or with deeper needle insertions. The statistical agreement between OpUS imaging and microscopic measurements of interstitial lesion depth, along with the previous study where the lesions were focused on the tissue surface [20], provide a first indication of the potential of OpUS for monitoring lesion formation and assessing treatment outcomes in different laser ablation applications. However, it should be noted that the small sample size of this study and visual bias in lesion depth measurement will impose limitations on statistical power.

OpUS exhibited good contrast (dynamic range: 35 dB) and imaging depth for B-mode images of the *ex vivo* kidney (Fig. 5). For both pre- and post-ablation images, the full depth of the kidney was visualised and the base could be seen beneath the tissue. For the pre-ablation image, the top surface of the kidney presented as a bright boundary with speckled contrast beneath. The calyx was visible at a depth of 10 mm, this was confirmed on microscope cross-sections of the kidney (Fig. 5). For the post-ablation image, two areas of higher brightness were visible, corresponding to the lesions formed during the LITT procedure (Fig. 5 (b)). However, the actual lesion size was difficult to measure from B-mode images, reflecting the significance of M-mode imaging.

Despite the promising accuracy and reliability of OpUS imaging for laser ablation monitoring, several modifications can be made for further pre-clinical studies. The imaging depth is valuable in clinical contexts to monitor the lesion formed in the deep-seated area. Whilst the imaging depth exceeded the maximum lesion size in this study, the depth could be increased further by increasing the generated ultrasound pressure. This could be achieved by increasing the excitation laser pulse energy or reducing the thickness of the CSNP composite coating [14,26]. However, care must be taken not to exceed the damage threshold of the coating material. For this study, the optical ultrasound transmitter and receiver worked inside a medical needle, where ultrasound reflections between the inner walls of the needle worsened the signal-to-noise ratio of the M-mode images. The cross-talk removal algorithm has reduced some of the reverberation artefacts. For future *in vivo* trials, ultrasonic attenuation materials can be coated on the inner wall of the needle to further mitigate this effect. Additionally, residual air in the needle lumen may cause impedance mismatch and affect the ultrasound transmission. In a clinical context, the device could be flushed with saline via a side arm adapter, which would eliminate any air within the needle and provide coupling for the probe. In this work, the laser light used for ablation was delivered through a bare optical fibre with a flat end face, creating lesions <1 cm in depth. As an alternative, it is common for LITT procedures to use diffuser tips to increase the area over which laser light is delivered [46]. Future studies can be carried out to assess the ability to monitor lesions formed with such diffuser tips and compare the resulting lesions between the two light delivery methods.

The work presented here demonstrates the feasibility of tracking LITT lesion formation in real-time with OpUS. The device developed had a small lateral profile ( <1 mm) and that permitted incorporation into a commercial-available medical needle, thereby potentially facilitating clinical translation. The good correlation between the OpUS measured lesion size and the post-ablation measurements opens the pathway to preclinical experiments for LITT lesion monitoring and represents a foundational step towards future clinical studies.

## Data Availability

Data underlying the results presented in this paper are not publicly available at this time but may be obtained from the authors upon reasonable request.
